# Circadian Behavioral Responses to Light and Optic Chiasm-Evoked Glutamatergic EPSCs in the Suprachiasmatic Nucleus of ipRGC Conditional vGlut2 Knock-Out Mice

**DOI:** 10.1523/ENEURO.0411-17.2018

**Published:** 2018-05-10

**Authors:** Michael G. Moldavan, Patricia J. Sollars, Michael R. Lasarev, Charles N. Allen, Gary E. Pickard

**Affiliations:** 1 Oregon Institute of Occupational Health Sciences, Oregon Health and Science University, Portland, OR 97239; 2 School of Veterinary Medicine and Biomedical Sciences, University of Nebraska, Lincoln, NE 68583; 3 Department of Behavioral Neuroscience, Oregon Health and Science University, Portland, OR 97239; 4 Department of Ophthalmology and Visual Sciences, University of Nebraska Medical Center, Omaha, NE 68198

**Keywords:** circadian rhythm, ipRGCs, melanopsin, retinohypothalamic tract, suprachiasmatic nucleus, vesicular glutamate transporter 2

## Abstract

Intrinsically photosensitive retinal ganglion cells (ipRGCs) innervate the hypothalamic suprachiasmatic nucleus (SCN), a circadian oscillator that functions as a biological clock. ipRGCs use vesicular glutamate transporter 2 (vGlut2) to package glutamate into synaptic vesicles and light-evoked resetting of the SCN circadian clock is widely attributed to ipRGC glutamatergic neurotransmission. Pituitary adenylate cyclase-activating polypeptide (PACAP) is also packaged into vesicles in ipRGCs and PACAP may be coreleased with glutamate in the SCN. vGlut2 has been conditionally deleted in ipRGCs in mice [conditional knock-outs (cKOs)] and their aberrant photoentrainment and residual attenuated light responses have been ascribed to ipRGC PACAP release. However, there is no direct evidence that all ipRGC glutamatergic neurotransmission is eliminated in vGlut2 cKOs. Here, we examined two lines of ipRGC vGlut2 cKO mice for SCN-mediated behavioral responses under several lighting conditions and for ipRGC glutamatergic neurotransmission in the SCN. Circadian behavioral responses varied from a very limited response to light to near normal photoentrainment. After collecting behavioral data, hypothalamic slices were prepared and evoked EPSCs (eEPSCs) were recorded from SCN neurons by stimulating the optic chiasm. In cKOs, glutamatergic eEPSCs were recorded and all eEPSC parameters examined (stimulus threshold, amplitude, rise time or time-to-peak and stimulus strength to evoke a maximal response) were similar to controls. We conclude that a variable number but functionally significant percentage of ipRGCs in two vGlut2 cKO mouse lines continue to release glutamate. Thus, the residual SCN-mediated light responses in these cKO mouse lines cannot be attributed solely to ipRGC PACAP release.

## Significance Statement

This study examined glutamatergic signaling by intrinsically photosensitive retinal ganglion cells (ipRGCs) of mice in which vesicular glutamate transporter 2 (vGlut2) was knocked out using Cre recombination. The results indicate that significant glutamatergic neurotransmission remains in ipRGCs of Opn4^Cre/+^;vGlut2^loxP/loxP^ mice and that the ipRGC vGlut2 conditional knock-out (cKO) model resulted in only subtle changes in the rate of vesicular glutamate replenishment even at high stimulation frequencies. These findings are consistent with the behavioral data observed in this and previous studies. Unfortunately, the residual ipRGC glutamatergic transmission in the Opn4^Cre/+^;vGlut2^loxP/loxP^ mouse model limits the usefulness of this model to examine the role of retinal peptidergic afferents to the suprachiasmatic nucleus (SCN) and also suggest caution when using Opn4^Cre/+^ mice in other Cre recombination models.

## Introduction

Retinal ganglion cells (RGCs), the projection neurons of the retina, transmit signals to a diverse group of subcortical target structures in the brain that contribute to image-forming and nonimaging functions (Morin and Studholme, 2014; Dhande et al., 2015). Targets of RGCs that mediate nonimage forming functions such as the hypothalamic suprachiasmatic nucleus (SCN), a circadian oscillator that functions as a biological clock, are innervated by a small subset of RGCs that express the photopigment melanopsin (Opn4). Melanopsin expression provides these retinal neurons the ability to depolarize and generate action potentials in direct response to photic stimulation (Berson et al., 2002; Hartwick et al., 2007). Two of the five currently recognized subtypes of melanopsin-expressing intrinsically photosensitive RGCs (ipRGCs; M1 and M2) send signals to the SCN via the retinohypothalamic tract (RHT; Berson et al., 2002; Hattar et al., 2002; Baver et al., 2008; Ecker et al., 2010; Estevez et al., 2012; Fernandez et al., 2016). These signals reset the SCN clock on a daily basis thereby entraining the circadian (i.e., ∼24 h) oscillation of clock gene expression and neural activity in the SCN to the 24-h day/night cycle (Sollars and Pickard, 2015).

A vast majority, if not all, RGCs convey signals to the brain using glutamatergic neurotransmission. This requires the packaging of glutamate into synaptic vesicles by one of the three isoforms of the vesicular glutamate transporter (vGlut; vGlut1–vGlut3) and ipRGCs use vGlut2 (Fyk-Kolodziej et al., 2004; Johnson et al., 2007; Kiss et al., 2008; Stella et al., 2008). In addition to glutamate, ipRGCs also sequester pituitary adenylate cyclase-activating polypeptide (PACAP) into synaptic vesicles, and PACAP may be coreleased with glutamate from RHT terminals in the SCN (Hannibal, 2006; Engelund et al., 2010). PACAP signaling may play a role in mediating some of the effects of light on the SCN and/or PACAP may modulate the effects of RHT glutamatergic neurotransmission, but the exact role of ipRGC PACAP neurotransmission in the SCN remains largely unknown.

To gain a better understanding of the potential role of PACAP in conveying ipRGC signals to the brain, a transgenic mouse strain was generated in which ipRGC glutamatergic neurotransmission was selectively impaired by eliminating the vGlut2 transporter in ipRGCs [ipRGC vGlut2 conditional knock-outs (cKOs); Delwig et al., 2013; Purrier et al., 2014; Gompf et al., 2015]. Behaviors dependent on ipRGC signaling (e.g., photoentrainment, pupillary light reflex, and neonatal photoaversion) were dramatically affected in the cKO mice, although not all ipRGC-mediated responses to light were completely eliminated. The residual ipRGC-mediated responses to light in the cKO animals have been attributed to the remaining ipRGC neurotransmitter, PACAP (Delwig et al., 2013; Purrier et al., 2014; Gompf et al., 2015; Keenan et al., 2016). However, the strength of this interpretation and ultimately the utility of the ipRGC vGlut2 cKO mouse strain are dependent on the extent to which glutamatergic neurotransmission has been eliminated in ipRGCs. Unfortunately, direct evidence showing that ipRGC glutamatergic neurotransmission is completely abolished in the ipRGC vGlut2 cKO mouse strain is lacking.

In the current study, we generated an ipRGC vGlut2 cKO mouse line similar to the mice described above. The Opn4-Cre mouse strain used in the studies described above in which Cre-recombinase is expressed in ipRGCs under control of the Opn4 promoter (Ecker et al., 2010) was crossed with a mouse strain in which the second exon of vGlut2 is flanked by loxP sites. In addition, we generated a second independent ipRGC vGlut2 cKO mouse line using a similar strategy of crossing a different mouse strain in which Cre-recombinase is expressed in ipRGCs under control of the Opn4 promoter (Hatori et al., 2008) with the same mouse strain in which the second exon of vGlut2 is flanked by loxP sites. Both vGlut2 cKO mouse lines were used to evaluate SCN-mediated behavioral responses to light. After behavioral data had been collected, a subset of animals was examined for RHT-mediated glutamatergic neurotransmission by recording EPSCs of SCN neurons evoked by optic chiasm stimulation in an *in vitro* slice preparation. The results from both ipRGC vGlut2 cKO mouse lines that we generated were similar and very clearly indicate that SCN-mediated responses to light are retained in almost all of these animals and that a functionally significant percentage of ipRGCs continue to release glutamate in the SCN. The results emphasize the need for physiologic verification of genetic mouse models and strongly undermine the interpretation that residual ipRGC-mediated behavior in ipRGC vGlut2 cKO mice is the result of light-evoked PACAP release from ipRGC terminals in the SCN.

## Materials and Methods

### Animals

Two mouse lines in which Cre-recombinase was knocked in to the Opn4 locus were used in this study. One mouse line described previously (Hatori et al., 2008) was generously provided by Satchidananda Panda (Salk Institute) and the other mouse line (Ecker et al., 2010) was generously provided by Samer Hattar (Johns Hopkins University). Mice from each line (referred to as Salk-Cre and Hopkins-Cre animals) homozygous for Cre (Opn4^Cre/Cre^) were crossed with mice homozygous for floxed-slc17a6 which encodes vGlut2 (these mice possess loxP sites flanking exon 2 of the vGlut2 gene; Slc17a6^tm1Lowl^/J, stock #012898, vGlut2^loxP/loxP^, The Jackson Laboratory). The F1 generation (Opn4^Cre/+^; vGlut2^loxP/+^) was backcrossed with vGlut2^loxP/loxP^ mice to generate cKOs (Opn4^Cre/+^; vGlut2^loxP/loxP^) and these mice were bred to generate animals lacking both melanopsin and vGlut2 [double KO (dKO); Opn4^Cre/Cre^; vGlut2^loxP/loxP^] and littermate controls (Opn4^+/+^; vGlut2^loxP/loxP^). It should be noted that in this breeding scheme: (1) the cKO animals retain a single copy of Opn4 and thus ipRGCs remain intrinsically photosensitive; and (2) the dKO mice should have no intrinsic photosensitivity remaining in ipRGCs as both copies of Opn4 should be replaced by Cre-recombinase. Animals were maintained under a light:dark (L:D) cycle consisting of 12-h 100-lux light followed by 12 h of complete darkness at 20–22°C with free access to food and water. All procedures were approved by the Institutional Animal Care and Use Committees and all efforts were made to minimize pain and the number of animals used.

### Behavioral studies

Mice were weaned at 21 d of age, separated by gender and maintained four animals per cage under 12/12 h L:D conditions until they were at least 8 weeks old. Mice of either gender were subsequently housed individually in cages equipped with running wheels under various lighting conditions and wheel-running behavior was recorded using ClockLab software (Actimetrics). Animal maintenance was performed with the aid of infrared night vision goggles (ITT-NE5001 generation 3, GT Distributors) when necessary. Three independent behavioral experiments using a total of 49 animals (16 littermate controls, 28 cKOs, and five dKOs) were conducted and electrophysiology was performed on 23 of the 49 mice. The free-running period was estimated using the last 10 d of activity under constant conditions.

#### Experiment 1

We report on behavioral data collected from 17 mice derived from the Salk-Cre mouse line (six littermate controls with one male and five females; eight cKOs with six males and two females; and three dKOs with one male and two female mice). Animals were maintained under LD 12:12 (100 lux:0 lux) for 106 d followed by 22 d in constant darkness (DD) followed by 61 d in constant light (LL; 100 lux). A cKO female animal died a few days before the termination of the study. None of these animals were used in electrophysiology experiments.

#### Experiment 2

This experiment used 12 mice derived from the Hopkins-Cre mouse line (six male littermate controls and six male cKOs). Animals were maintained under LD 12:12 (100 lux:0 lux) for 84 d followed by 114 d in DD followed by 73 d in LD 12:12 (1000 lux:0 lux). At the completion of behavioral data collection, physiologic recordings of SCN neurons in an *in vitro* slice preparation were made from six animals (five littermate controls and one cKOs).

#### Experiment 3

This experiment used 20 mice; 10 derived from the Salk-Cre mouse line and 10 derived from the Hopkins-Cre mouse line. Of the Salk-Cre animals, three were littermate controls (one male and two females), five were cKOs (three males and two females), and two were dKOs (one male and one female). Of the Hopkins-Cre animals, there was one male littermate control and nine were cKOs (four males and five females). Animals were maintained under LD 12:12 throughout this experiment. Five animals were housed under 1000 lux:0 lux for 35 d followed by 100 lux:0 lux for 22 d. The other 15 animals were housed under 100 lux:0 lux for 37 d followed by 1000 lux:0 lux for 40 d. At the completion of behavioral data collection, physiologic recordings of SCN neurons in an *in vitro* slice preparation were made from 17 of these animals (five controls, 10 cKOs, and two dKOs). In addition, recordings were made from adult wild-type (WT) mice (*n* = 16; C57BL/6J, male, The Jackson Laboratory).

### Animal housing and brain slice preparation

Before *in vitro* brain slice recording, all male and female mice (six months old and older) were maintained at 20–21°C on a 12/12 h L:D cycle (light onset 6 A.M., zeitgeber time 00:00) in an environmental chamber (Percival Scientific), with free access to food and water for a minimum of six weeks. The mice were deeply anesthetized with isoflurane (Hospira, Inc) during the light phase, and brains were removed and submerged in ice-cold Krebs slicing solution consisting of: 82.7 mM NaCl, 2.4 mM KCl, 0.5 mM CaCl_2_, 6.8 mM MgCl_2_, 1.4 mM NaH_2_PO_4_, 23.8 mM NaHCO_3_, 23.7 mM dextrose, and 60 mM sucrose, saturated with 95% O_2_ and 5% CO_2_; pH 7.3–7.4, 308–310 mOsm. Coronal (200–250 µm) hypothalamic brain slices containing the SCN were cut with a vibrating-blade microtome (Leica VT 1000 S, Leica Biosystems GmbH). Slices were incubated in the slicing solution 1–1.5 h at 30°C before electrophysiological recordings were initiated.

### Whole-cell patch clamp recording

Recordings were made at 28°C using the whole-cell patch-clamp technique from 1.5 to 8 h after slice preparation. The superfusion solution was warmed with a heater (Model SH-27B Inline Heater, Warner Instruments Corp.) just before the solution entered the recording chamber. The bath temperature in the recording chamber was monitored continuously with a thermistor probe, which provided feedback to a dual automatic temperature controller (TC-344B, Warner Instruments Corp.). The recording solution was: 132.5 mM NaCl, 2.5 mM KCl, 1.2 mM NaH_2_PO_4_, 2.4 mM CaCl_2_, 1.2 mM MgCl_2_, 11 mM glucose, and 22 mM NaHCO_3_, saturated with 95% O_2_ and 5% CO_2_; pH 7.3–7.4, 300–305 mOsm. Microelectrodes with resistances of 7–9 MΩ were pulled from borosilicate glass capillaries (World Precision Instruments, Inc.) and filled with a solution containing: 115 mM CH_3_O_3_SCs, 8 mM CsCl, 0.5 mM CaCl_2_, 10 mM HEPES, 5 mM EGTA, 13 mM CsOH, 3 mM MgATP, 0.3 mM TrisGTP, and 5 mM N-(2,6-dimethylphenylcarbamoylmethyl)triethylammonium chloride (QX-314); pH 7.25, 278 mOsm. Lidocaine N-ethyl chloride (QX-314) was included in the patch pipette solution to block voltage-dependent Na^+^ currents. Cs^+^ was used to block postsynaptic K^+^ channels including GABA_B_-activated K^+^ channels (Jiang et al., 1995). With applied internal solution the equilibrium potential for chloride was -60 mV, which substantially decreased or virtually eliminated GABA_A_ receptor-mediated currents. Additionally, the external solution contained picrotoxin (50 µM) and (2S)-3-[[(1S)-1-(3,4-dichlorophenyl]amino-2-hydroxypropyl](phenylmethyl)phosphinic acid (CGP55845; 3 µM) to prevent activation of GABA_A_ and GABA_B_ receptors. To confirm glutamatergic RHT neurotransmission, the AMPA receptor antagonist 6-cyano-7-nitroquinoxaline-2,3-dione (CNQX; 20 µM) was applied at the end of each recording. Individual SCN neurons were visualized with infrared illumination and differential interference contrast optics using a Leica DMLFS (Leica Biosystems GmbH) microscope with video camera and display (Sony). On-line data collection and analysis were performed using an EPC-7 patch-clamp amplifier (HEKA Electronik), a Mac mini-computer and Patchmaster software (HEKA Electronik). The records were filtered at 3 kHz and digitized at 10 kHz.

To allow equilibration between the pipette solution and the cell cytoplasm, whole-cell voltage-clamp recording started ∼10 min after rupturing the membrane. A small hyperpolarizing voltage step (-2 mV, 5 ms) was applied before optic chiasm stimulation to monitor the series resistance, which was not compensated. SCN neurons were voltage-clamped at -60 mV. During the recording the series resistance remained stable and the recordings with series resistance changes of <10% were included in the data analysis. Slow and fast capacitances were not compensated.

### Optic chiasm stimulation

EPSCs were evoked by electrical stimulation of the optic chiasm with a Grass S88 stimulator (Grass Medical Instruments) using a concentric bipolar tungsten electrode (outer pole diameter 0.125 µm; catalog #CBASC75, FHC) connected to a stimulus isolation unit (model SIU5B, Grass Medical Instruments). The stimulating electrode was positioned in the middle of the optic chiasm as far from the SCN as possible. The stimulus pulse duration was 0.13–0.17 ms, and stimulation intensity was set 1.5–2.0 times higher than that needed to evoke a threshold response. To evoke EPSCs a single stimulus or stimulus trains were applied. To determine the threshold stimulus needed to evoke the EPSC, and to study the dependence of the evoked EPSC (eEPSC) amplitude on the strength of applied stimulus, the voltages that were used to stimulate the optic chiasm were applied in the range of 3–30 V. EPSCs were also evoked by trains of 25 stimuli (square pulses) separated by 30-s intervals at frequencies 0.08, 0.5, 1, 2, 5, 10, and 25 Hz. Three stimulus trains were applied for each frequency/recorded neuron. The inclusion of ion channel blockers in the internal solution as well as voltage-clamping at -60 mV prevented the activation of voltage-dependent ionic currents in SCN neurons.

### Test agent application

All test agents were bath applied in artificial CSF (ACSF) containing the final concentration of the compounds. A complete change of the external solution took <30 s at a flow rate of 1.5–2 ml/min. Picrotoxin, CNQX, and QX-314 were purchased from Sigma, and CGP55845 was obtained from Tocris Cookson Inc. Appropriate stocks were made and diluted with ACSF just before application. To make stocks, CNQX and CGP55845 were dissolved in dimethyl sulfoxide (DMSO; the final concentration of DMSO in ASCF was 0.01%), and picrotoxin was dissolved in ethanol (the final concentration of ethanol in ASCF was 0.1%).

### Statistical analysis

Stimulation of the optic chiasm at 0.08 Hz does not induce synaptic depression at RHT-SCN synapses, and the eEPSC amplitude is stable for long durations (Moldavan and Allen, 2010; Moldavan et al., 2010, 2013). The stimulus amplitude to activate an eEPSC, the maximal eEPSC amplitude, the series resistance and the following parameters of eEPSCs were analyzed: amplitude, time to the peak (a delay between the stimulus artifact and the peak of eEPSC), rise time (10–90% of amplitude), and decay time constant. There were 25 eEPSCs recorded from each neuron, and some slices had more than one neuron recorded and there were three mouse genotypes. Because of this data clustering and nesting, we analyzed the eEPSC data using generalized estimating equation (GEE; γ or Gaussian distribution, with exchangeable correlation structure and robust sandwich estimates of SE) using the statistical program R (version 1.6.1 with nonlinear and linear mixed effects models package version 3.1–3.6; obtained from http://cran.r-project.org; R Core Team, 2017). A Kruskal–Wallis rank sum test was used to test the deviation of the eEPSC amplitudes. During application of long stimulus trains (0.5–25 Hz, 25 stimuli in each train), synaptic depression caused the eEPSC amplitude to decrease to a steady state (plateau). eEPSC amplitude was measured as the difference between the peak eEPSC current and the baseline level before the stimulus artifact. To compare synaptic depression under different conditions and between recorded neurons the amplitude of each subsequent eEPSC (eEPSCn) during repetitive stimulation was normalized (in %) to the amplitude of the first eEPSC (eEPSC_1_) in the stimulus train: ratio eEPSCn/eEPSC_1_. To take into account the variability of the eEPSC amplitude the mean amplitude of the first eEPSC was calculated from three stimulus trains at each stimulus frequency for each neuron. The normalized amplitudes were averaged across all recorded neurons (n), presented as the mean ± SEM and plotted against stimulus number in the train. Extra sum of squares *F* test was used to compare the datasets recorded under different conditions. Two-sample assuming unequal variances two-tailed *t* tests were used to compare the control and test data for each dataset (for each stimulus frequency). A confidence level of 95% was used to determine statistical significance.

Igor Pro 5.03 (Wave Metrics, Inc.), KaleidaGraph TM 3.6 (Synergy Software), Excel 11.6.6 (or 14.4.5; Microsoft Co), FreeHand MX (Macromedia, Inc), and R (http://cran.r-project.org) were used for curve fitting, data analysis, and graphic presentation. The spontaneous EPSCs were analyzed using MiniAnalysis (Synaptosoft, Inc.).

## Results

### Experiment 1. Wheel-running behavior of Salk-Cre vGlut2 KO mice

Wheel running behavior of 17 mice derived from the Salk-Cre mouse line (six littermate controls, eight cKOs, and three dKOs) was examined under LD 12:12 (100 lux:0 lux; 106 d) followed by DD (0 lux; 22 d) followed by LL (100 lux; 61 d) conditions. No gender differences were noted in the behavior of the animals.

#### Littermate controls (Opn4^+/+^; vGlut2^loxP/loxP^)

**Figure 1.**
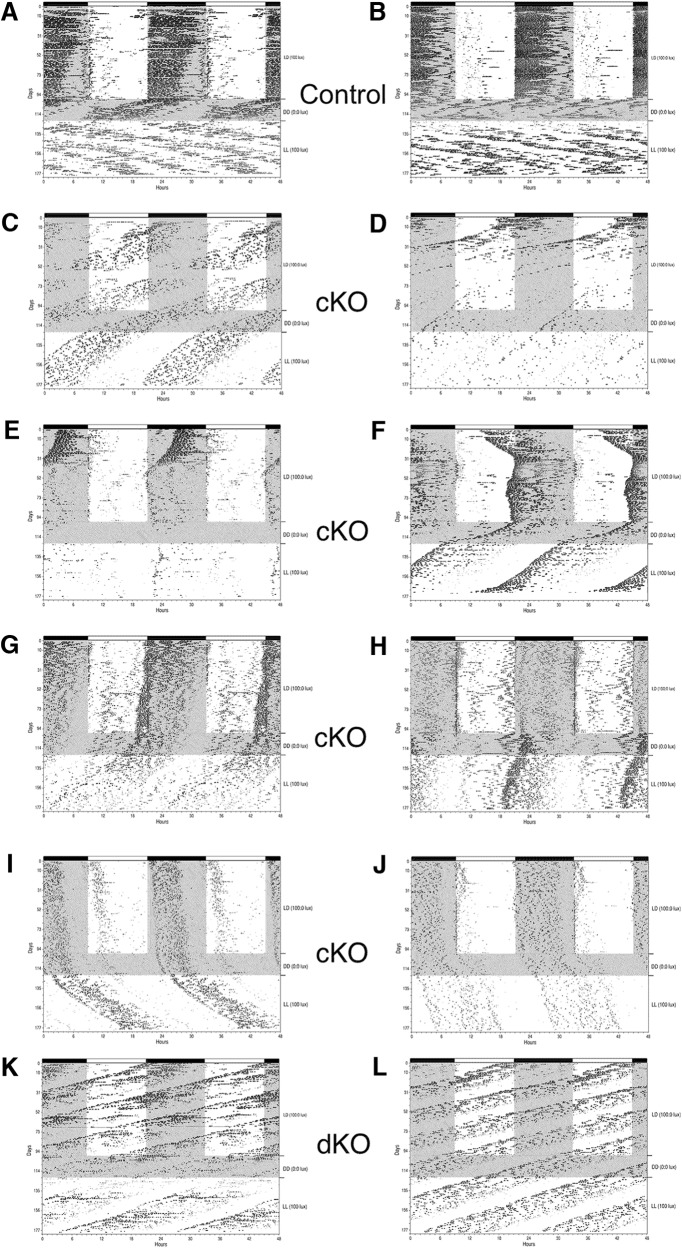
Wheel-running behavior of Salk-Cre mice. Wheel-running activity records (double-plotted) of control (Opn4^+/+^; Vglut2^loxP/loxP^; ***A***, ***B***), cKO (Opn4^Cre/+^; Vglut2^loxP/loxP^; ***C–J****)*, and dKO (*Opn4^Cre/Cre^; Vglut2^loxP/loxP^*; ***K***, ***L***) mice generated from the Salk-Cre line maintained under L12:D12 (100 lux:0 lux) for 106 d followed by DD for 22 d and LL (100 lux) for 61 d.

The six littermate control mice entrained to the LD cycle with activity onsets at or near light offset as expected (representative examples are provided in Fig. 1*A*,*B*). Under DD conditions, only five of the six animals had activity levels or activity onsets robust enough for analysis. The free-running period (τ) in DD (τ_DD_) ranged from 23.3 to 24.1 h (23.70 ± 0.14 h, *n* = 5, mean ± SEM). Under LL 100 lux (LL_100_) conditions, τ lengthened in all animals as expected; τ_LL_ was greater than τ_DD_ for each animal and ranged from 24.7 to 25.8 h. The mean τ_LL_ was significantly greater than mean τ_DD_ for the controls (25.28 ± 0.17 h vs 23.70 ± 0.14 h, *n* = 5; *p* < 0.001; Fig. 1*A*,*B*). In summary, SCN-mediated behavior was typical of mice with functioning ipRGCs and of mice with a mixed genetic background.

#### cKOs (Opn4^Cre/+^; vGlut2^loxP/loxP^)

Entrainment under the LD 12:12 conditions was abnormal in seven of the eight cKO animals. Two cKO animals appeared to free-run under LD conditions (Fig. 1*C*,*D*). Two other cKO animals initially appeared to be free-running under the LD 12:12 conditions but were apparently very gradually entraining to the LD cycle (Fig. 1*E*,*F*). These animals required one to two months before wheel-running onsets were somewhat “aligned” to light offset. Activity onsets remained near light offset from that time forward but entrainment was unstable with activity onsets of one mouse gradually advancing to a phase angle of entrainment of between 1 and 2 h before light offset. The activity onsets of the other mouse slowly drifted away from light offset such that when the LD cycle was terminated and animals entered DD conditions, activity onsets were initiated approximately 1 h after light offset (Fig. 1*E*,*F*). Three of the cKOs demonstrated activity onsets that drifted over time under LD conditions. One cKO animal had activity onsets that gradually advanced as time went on resulting in a positive 2-h phase angle of entrainment (Fig. 1*G*). Another cKO had very unstable and erratic behavior with a positive phase angle of entrainment (Fig. 1*H*). The other cKO had stable activity onsets that were initiated ∼30 min after light offset, but the activity onsets gradually drifted to ∼2 h after light offset at the time animals went into DD (Fig. 1*I*). The last cKO in this group had relatively stable activity onsets initiated at light offset throughout the LD cycle portion of the experiment (Fig. 1*J*).

The cKO animals that showed some level of entrainment under LD conditions free-ran in DD with activity onsets initially aligned with the onsets in the prior LD cycle indicating there was no masking of activity onsets in the prior LD cycle. Activity levels permitted an estimate of the free-running period in DD for seven cKO animals and τ_DD_ ranged from 23.3 to 24.2 h; the mean cKO τ_DD_ was not significantly different from the τ_DD_ of the controls (cKO, 23.83 ± 0.12 h, *n* = 7 vs controls 23.70 ± 0.14 h, *n* = 5; *p* = 0.50).

Six cKOs had sufficient wheel-running activity to estimate the free-running period in LL. Of these six animals, τ_LL_ was slightly greater than τ_DD_ in two cases, unchanged in one case, and slightly decreased in three animals. Thus, for the cKOs as a group, the mean τ_LL_ was similar to the mean τ_DD_ (DD, 23.83 ± 0.12 h vs LL, 23.90 ± 0.11 h; *p* = 0.668) and almost 1.5 h less than the mean τ_LL_ of controls (23.90 ± 0.11 vs 25.28 ± 0.17; *p* < 0.0001).

In summary, this small cohort of cKO animals demonstrated a wide range of ipRGC-mediated responses to light. The circadian behavioral responses to light ranged from animals with virtually no response to light (i.e., free running behavior throughout all lighting conditions) to entrainment with abnormal phase angles to an animal with virtually normal entrainment.

#### dKOs (Opn4^Cre/Cre^; vGlut2^loxP/loxP^)

Two of the three dKO animals appeared to free-run with a period of <24 h under all lighting conditions with a slight lengthening of τ (≈0.1–0.2 h) under LL conditions (Fig. 1*K*,*L*). The third dKO mouse also appeared to free-run under all conditions although activity onsets were more erratic and there appeared to be a gradual lengthening of τ over the course of the experiment (data not shown). The behavior of these animals lacking melanopsin and vGlut2 in ipRGCs suggested that there was very little behavioral response to light.

### Experiment 2. Wheel-running behavior of Hopkins-Cre vGlut2 cKO mice

Wheel running behavior of 12 animals (six littermate controls and six cKO) derived from the Hopkins-Cre mouse line was examined under LD 12:12 (100 lux:0 lux; 84 d) followed by DD (114 d) followed by LD (1000 lux:0 lux; 74 d) conditions.

#### Littermate controls (Opn4^+/+^; vGlut2^loxP/loxP^)

**Figure 2.**
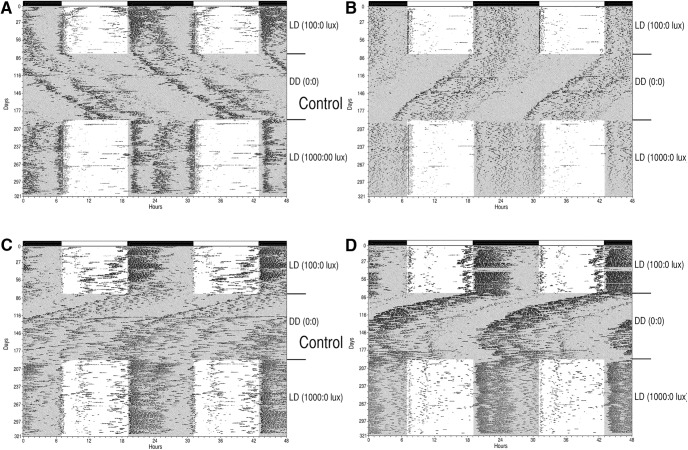
Wheel-running behavior of Hopkins-Cre mice. Wheel-running activity records (double-plotted) of representative Control mice (Opn4^+/+^; Vglut2^loxP/loxP^; ***A–D***) generated from the Hopkin-Cre line maintained under L12:D12 (100 lux:0 lux) for 84 d followed by DD for 114 d and then L12:D12 (1000 lux:0 lux) for 73 d.

Of the littermate control animals, five entrained to the LD cycle with activity onsets at or near light offset as expected although there was a variable level of activity present during the light phase in most of the animals (Fig. 2*A–D*). One littermate control animal had diffuse and erratic wheel-running behavior and is not considered further. Under the prolonged DD conditions, four of the five remaining littermate control animals had activity that free-ran; one mouse stopped running in the wheel after a few weeks in DD and is not considered further. The free-running activity was stable for two of the animals during DD with periods of 24.2 and 23.9 h (Fig. 2*A*,*B*), whereas the free-run became less stable over time for one mouse (τ_DD_ = 23.7 h; Fig. 2*C*) while another animal began free-running in DD with a short period (τ_DD_ = 23.5 h; Fig. 2*D*), which changed over time to be become >24 h by the termination of DD conditions. These four littermate control animals rapidly reentrained to the brighter LD cycle (1000 lux:0 lux) with phase angles of entrainment similar to those under the original (100 lux:0 lux) LD cycle. The animals generally demonstrated less activity during the 1000-lux light phase compared to their activity levels during the dimmer 100-lux light phase.

#### cKOs (Opn4^Cre/+^; vGlut2^loxP/loxP^)

**Figure 3.**
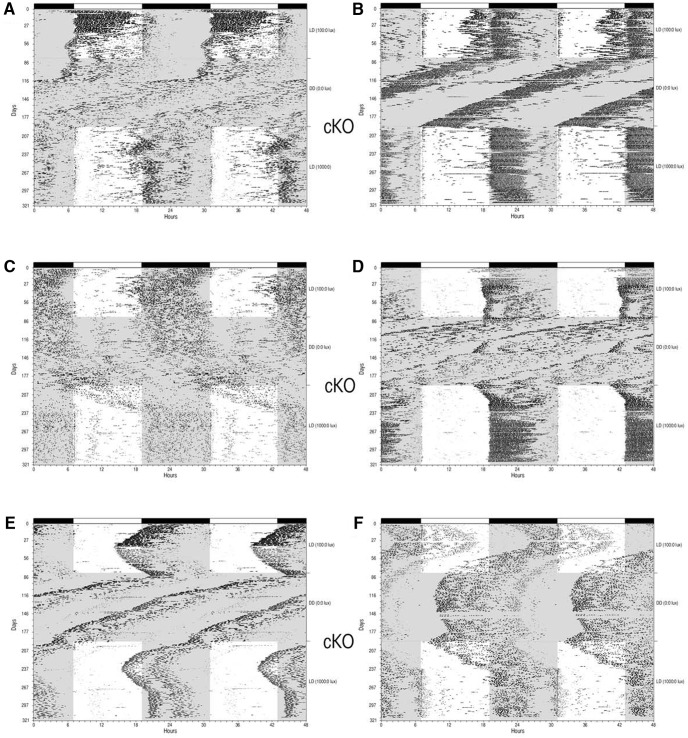
Wheel-running behavior of Hopkins-Cre mice. Wheel-running activity records (double-plotted) of cKO mice (Opn4^Cre/+^; Vglut2^loxP/loxP^; ***A–F***) generated from the Hopkins-Cre line maintained under L12:D12 (100 lux:0 lux) for 84 d followed by DD for 114 d and then L12:D12 (1000 lux:0 lux) for 73 d.

The six cKO animals demonstrated a range of aberrant behavior under the initial 12:12 100 lux:0 lux LD cycle (Fig. 3*A–F*). One mouse had an extremely large positive phase angle of entrainment (≈12 h) with wheel running initiated around light onset. During the 84 d under LD, 100 lux:0 lux onsets gradually advanced further with wheel-running onsets beginning during the end of the dark phase although onsets were quite variable (Fig. 3*A*). Three mice also demonstrated a positive phase angle of entrainment of several hours (i.e., wheel-running was initiated during the light phase of the LD cycle), but due to the variability in the onsets, a precise phase angle was not estimated (Fig. 3*B–D*). The remaining two mice never entrained to the initial LD 100 lux:0 lux, although it was clear that the LD cycle was impacting their behavior as evidenced when transferred to DD (Fig. 3*E*,*F*). All mice free-ran under DD conditions (Fig. 3*A–F*). When a bright 12L:12D cycle was reinitiated (1000 lux:0 lux) following 114 d in DD, all animals responded by shifting their wheel-running onsets toward light offset. Four animals entrained to the bright LD cycle with a less positive phase angle compared to their entrainment under the dimmer LD cycle and in three cases onsets were initiated in the dark (Fig. 3*A–D*). The two animals that did not entrain to the initial LD cycle looked as if they might entrain to the bright LD cycle when the experiment was terminated following 74 d in LD 1000 lux:0 lux (Fig. 3*E*,*F*).

In summary, all six cKOs showed residual SCN-mediated responses to light and entrainment was generally improved under the brighter lighting conditions, suggesting that enduring circadian responses to light were facilitated by the increase in environmental luminance.

### Experiment 3. Wheel-running behavior of Salk-Cre and Hopkins-Cre vGlut2 KO mice

Wheel running behavior of 20 animals (10 derived from the Hopkins-Cre mouse line and 10 derived from the Salk-Cre line) was examined. Five animals (four Salk-Cre and one Hopkins-Cre) were housed under 1000 lux:0 lux for 35 d followed by 100 lux:0 lux for 22 d. The other 15 animals (six Salk-Cre and nine Hopkins-Cre) were maintained under 100 lux:0 lux for 37 d followed by 1000 lux:0 lux for 40 d.

#### Littermate controls (Opn4^+/+^; vGlut2^loxP/loxP^)

**Figure 4.**
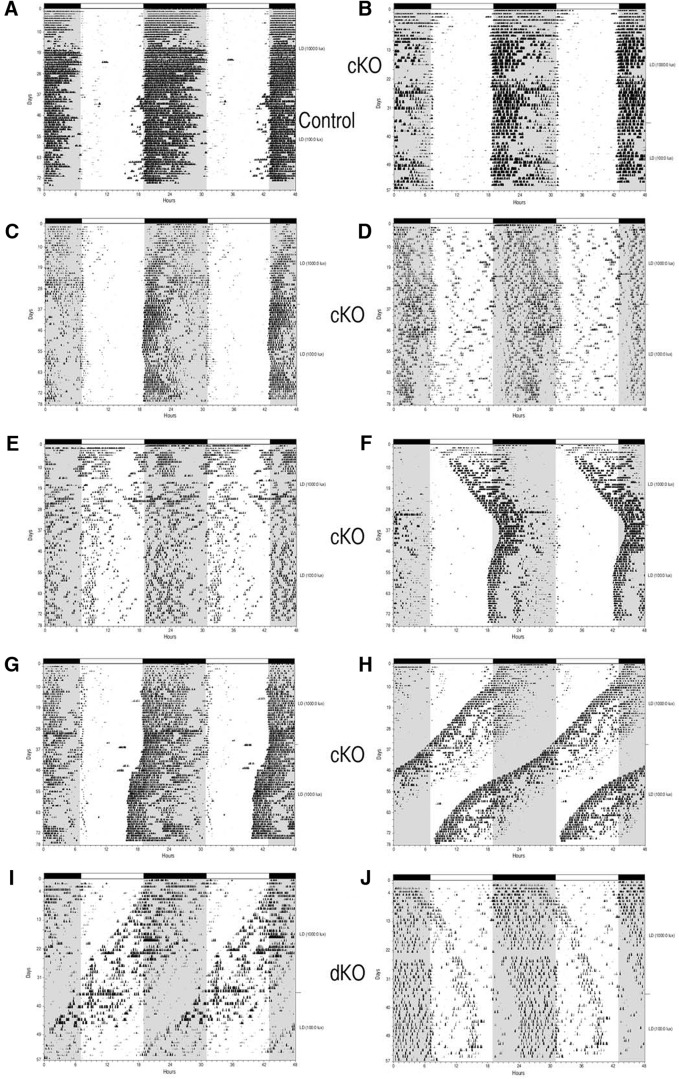
Wheel-running behavior of Salk-Cre and Hopkins-Cre mice. Wheel-running activity records (double-plotted) of Control (Opn4^+/+^; Vglut2^loxP/loxP^; **A**), cKO (Opn4^Cre/+^; Vglut2^loxP/loxP^; ***B–H***) and dKO *(Opn4^Cre/Cre^; Vglut2^loxP/loxP^*; ***I***, ***J***) mice maintained under L12:D12 conditions. Animals shown in ***A***, ***C–H*** were maintained initially under 100 lux:0 lux for 37 d followed by 1000 lux:0 lux for 40 d. Animals shown in ***B***, ***I***, ***J*** were maintained initially under 1000 lux:0 lux for 35 d followed 100 lux:0 lux for 22 d.

Four littermate control animals were studied: three Salk-Cre and one Hopkins-Cre. All of the animals entrained to the initial 100 lux:0 lux LD cycle with onsets initiated at light offset as expected; changing the illuminance level to 1000 lux during the light phase had very little impact on their wheel-running activity. A representative control animal is illustrated in Figure 4*A*.

#### cKOs (Opn4^Cre/+^; vGlut2^loxP/loxP^)

Three cKO animals (two Salk-Cre and one Hopkins-Cre) began the experiment in the 1000 lux:0 lux LD cycle. These animals entrained with activity onsets at or very near light offset similar to control animals and as observed in the controls, changing illuminance levels for these three animals from 1000 lux to 100 lux had little impact on their pattern of entrainment to the LD cycle as shown by a representative example in Figure 4*B*. The remaining 11 cKO animals (three Salk-Cre and eight Hopkins-Cre) began the experiment under 100 lux:0 lux LD conditions. One of these animals demonstrated a normal pattern of entrainment with activity onsets initiated near light offset throughout both LD cycles (Fig. 4*C*). Wheel-running activity was aberrant in the other 10 mice. Several animals appeared to entrain to the LD cycle but with: (1) an altered phase angle under both lighting conditions (Fig. 4*D*); (2) clear entrainment only under the brighter LD cycle (Fig. 4*E*,*F*); and (3) a dramatic change in the phase angle of entrainment when the illuminance was changed from 100 lux to 1000 lux (Fig. 4*G*). Two cKOs did not entrain, although light did impact their wheel-running behavior, which is best described as an oscillatory free-run (Fig. 4*H*). In summary, the behavior of the cKOs in this experiment ranged from apparent normal entrainment to completely un-entrained and free-running but not completely unresponsive to light (i.e., oscillatory free-run).

#### dKOs (Opn4^Cre/Cre^; vGlut2^loxP/loxP^)

Two dKO animals (Salk-Cre) were examined in this experiment. First, in LD 1000 lux:0 lux and then in LD 100 lux:0 lux conditions. One mouse free-ran throughout the study with light having little impact on the wheel-running activity (Fig. 4*I*). The other dKO mouse appeared to free-run initially under 1000 lux:0 lux conditions but then activity onsets stabilized under 100 lux:0 lux conditions (Fig. 4*J*).

### RHT-evoked EPSCs in SCN neurons

After behavioral testing was completed in experiments 2 and 3, 23 of the 32 animals were used to examine RHT-SCN synaptic transmission. For these studies brain slices were prepared for *in vitro* recording of glutamate-mediated EPSCs in SCN neurons. We hypothesized that glutamate release from RHT axonal terminals would be eliminated in the SCN of vGlut2 cKO mice, and thus the EPSC evoked by optic chiasm stimulation would be abolished and the sEPSC frequency would be greatly reduced.

**Figure 5.**
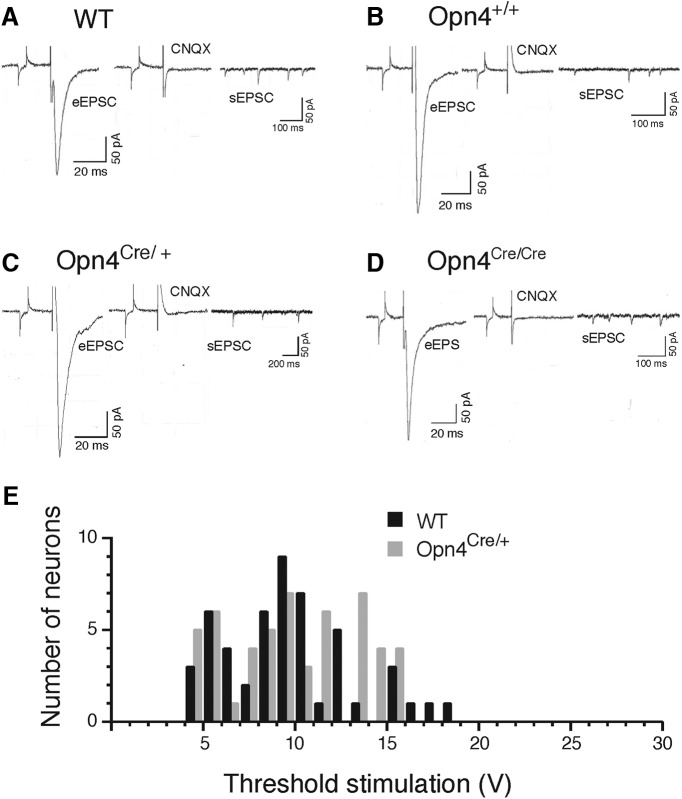
Evoked and spontaneous EPSCs in SCN neurons. Voltage-clamp recordings of eEPSCs in SCN neurons of (***A***) WT; (***B***) control (Opn4^+/+^; Vglut2^loxP/loxP^); (***C***) cKO (Opn4^Cre/+^; Vglut2^loxP/loxP^); and (***D***) dKO (Opn4^Cre/Cre^; Vglut2^loxP/loxP^) mice. EPSCs (membrane potential clamped at -60 mV) were evoked by stimulation of the optic chiasm in the absence of CNQX (left recording in ***A–D***) and in the presence of AMPA/kainate antagonist CNQX (20 µM, right recording in ***A–D***). Each recording in ***A–D*** shows a test current followed by a stimulus artifact and the eEPSC. sEPSC are shown in the recordings on the right. The corresponding actograms for each of the mice (***B***, ***C***) are shown in Figure 4*B* (***B***), *G* (***C***), and *I* (***D***). ***E***, A histogram showing the distribution of the stimulation voltage required to evoke the threshold EPSC in WT (*n* = 50 neurons, black bars) and cKO mice (*n* = 52, gray bars); there was no significant difference between the two groups.

First, we confirmed that the EPSCs evoked by optic chiasm stimulation were mediated by RHT glutamate release. All eEPSCs recorded in SCN neurons from WT animals and littermate controls were eliminated by bath application of the AMPA receptor antagonist CNQX (20 µM) indicating that EPSCs evoked by optic chiasm stimulation require activation of glutamatergic AMPA receptors (Fig. 5*A*,*B*). Stimulation of the optic chiasm also evoked EPSCs in vGlut2 cKOs and dKOs and these EPSCs were also eliminated by application of CNQX (Fig. 5*C*,*D*). The finding that optic chiasm stimulation evoked glutamatergic EPSCs in vGlut2 KOs was unexpected. We had anticipated that no glutamatergic eEPSCs would be observed following optic chiasm stimulation in animals in which vGlut2 expression had been eliminated in ipRGCs.

The observed RHT-mediated eEPSCs in vGlut2 cKO and dKO mice might have been the result of only a partial rather than a complete block of glutamate packaging into ipRGC synaptic vesicles. If this were the case, stronger stimuli might be required to evoke an EPSC. To address this possibility, we studied the stimulus strength required to evoke an EPSC in vGlut2 cKO mice. In this experiment the optic chiasm was stimulated with a single pulse and the stimulus intensity was gradually increased in 1-V steps from 3 to 30 V. The recordings from cKO mice (*n* = 52 neurons) were compared with recordings from WT controls (*n* = 50 neurons). The distribution of stimulus voltages which evoked threshold EPSCs was similar for WT and cKO (*p* = 0.310, GEE; Fig. 5*E*; Table 1). The thresholds also were similar for WT versus controls and cKO versus controls (*p* = 0.864 and *p* = 0.518, respectively, GEE; Table 1). These results indicate that glutamate filling of synaptic vesicles was not substantially altered in cKO mice.

**Table 1.** Parameters of eEPSCs in SCN neurons

Mouse genotype	WT	Controls	cKO	**p** value
Number of mice	16	9	12	
Neurons/eEPSC	19/475	20/500	16/377	
Peak amplitude (pa)	_156.3_ 187.8 _225.7_	_139.7_ 181.4 _235.6_	_143.2_ 170.6 _203.3_	0.756
Time to peak (ms)	_4.6_ 5.1 _5.5_	_4.5_ 4.8 _5.1_	_4.4_ 4.9 _5.5_	0.636
Rise time (ms)	_1.3_ 1.53 _1.7_	_1.3_ 1.5 _1.8_	_1.2_ 1.5 _1.9_	0.815
Decay time constant (ms)	_5.1_ 6.4 _7.6_	_4.8_ 5.4 _6.0_	_4.0_ 4.9 _5.8_	0.173
Threshold (V)	_7.6_ 9.0 _10.4_	_6.9_ 8.8 _10.7_	_6.8_ 8.0 _9.3_	0.569
Stimulation (V)	_12.1_ 13.5 _14.9_	_11.8_ 14.3 _16.9_	_14.4_ 16.7 _18.9_	0.065

WT: wild-type controls; controls: littermate controls; cKO: Opn4^Cre/+^; Vglut2^loxP/loxP^. LL and UL CI: lower and upper limits of confidence interval; GEE was used to calculate means, confidence intervals, and *p* values (shown for comparison between all three mouse types). The center value indicates the mean. The subscripted values indicate the lower (left) and upper (right) limits of the 95% confidence interval. EPSCs were evoked by 0.08-Hz stimulation of the optic chiasm, which did not induce synaptic depression.

**Figure 6.**
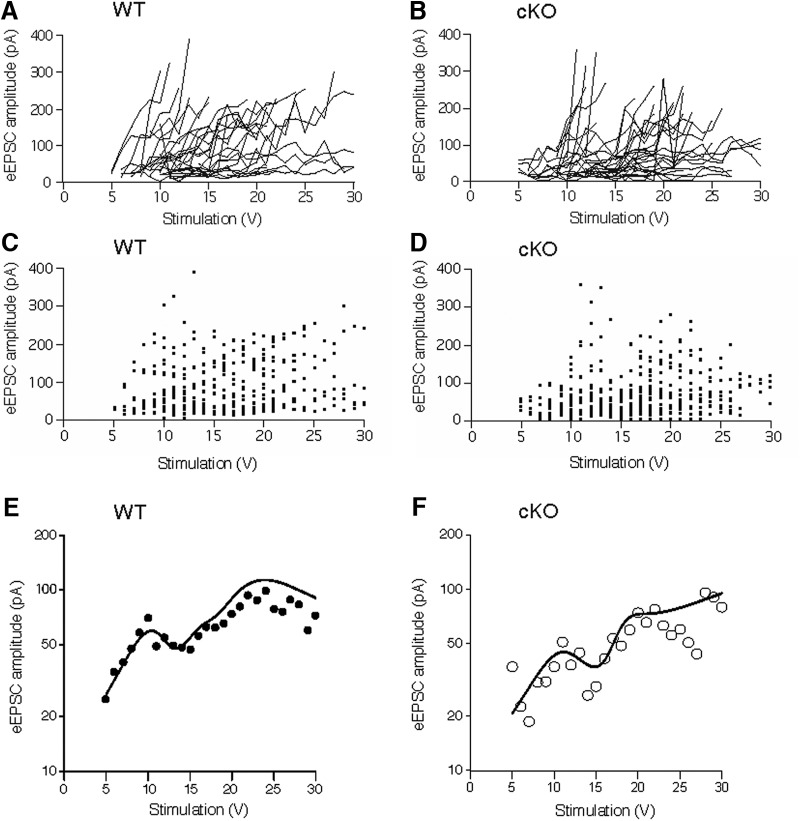
The relationship between eEPSC amplitude and stimulus strength in WT and cKO SCN neurons. Dependence of eEPSC amplitude (pA) on the strength of stimulus (V) applied to the optic chiasm in WT and Opn4^Cre/+^ mice (***A–F***). ***A***, ***C***, ***E***, WT (*n* = 35 SCN neurons). ***B***, ***D***, ***F***, cKO (Opn4^Cre/+^; Vglut2^loxP/loxP^; *n* = 41 SCN neurons). ***A***, ***B***, Each line on the graph represents voltage-dependent changes of eEPSC for an individual neuron. ***C***, ***D***, Scatter histograms showing the distribution of EPSC amplitudes evoked by stimulation. ***E***, ***F***, The results of each fitted linear mixed-effect model (lines) were used to analyze the eEPSC amplitude (pA) model at the population level (averaged over all animal-specific random effects) for the WT and Opn4^Cre/+^ mice (***E***, ***F***). The plotting symbols show the geometric mean amplitude at each stimulation amplitude. Error bars are omitted as there is no unique or unambiguous way to define SE when multiple sources of variation are present.

Another potential explanation for the unexpected observation of optic chiasm stimulation-evoked EPSCs in SCN neurons of cKO mice might have been the result of a failure to eliminate glutamate packaging in all ipRGCs that innervate the SCN resulting in some ipRGCs transmitting signals normally. If only some ipRGCs afferent to the SCN were capable of releasing glutamate normally, then a stronger stimulus might be required to evoke a maximal response in SCN neurons. To explore this possibility, the relationship between the eEPSC amplitude and the stimulus strength was compared between SCN neurons in WT controls and cKOs animals (Fig. 6). The SCN neuronal responses observed in both WT control and cKO animals were highly variable. Some recorded neurons showed gradually increasing eEPSC amplitudes with increasing stimulus intensities whereas in other SCN neurons, the eEPSC amplitude rapidly reached the maximal level during increasing stimulation intensities (Fig. 6*A*,*B*). In these neurons, the maximal eEPSC amplitude was observed when the stimulus strength exceeded 1.5–2.0 times the threshold level. Linear mixed-effect models were used to analyze eEPSC amplitude (pA) as a function of stimulus (V). To account for nonlinear behavior, stimulation (range: 3–30 V) was decomposed using restricted cubic splines (RCS) with seven knots. (Knot placement corresponded roughly to the 2.5th, 18.3th, 34.2th, 0.50th, 0.66th, 0.82th, and 97.5th percentiles of stimulus.) Models treated an individual mouse as a random effect. Influence of genotype (WT vs cKO) was assessed through a test of the all RCS components interacting with genotype. There was no evidence to suggest eEPSC amplitude as a function of stimulus was modified by genotype [X^2^ (6 df) = 2.66, *p* = 0.85; test of RCS:genotype interactions]. Further, there was no indication that genotype was associated with eEPSC amplitude [X^2^ (7 df) = 4.69, *p* = 0.70; composite geno + RCS:geno]. Figure 6*A*,*B* shows results from each fitted model at the population level (averaged over all animal-specific random effects). Plotting symbols show the geometric mean amplitude at a given stimulation. Error bars are omitted as there is no unique or unambiguous way to define SE when multiple sources of variation are present.

**Figure 7.**
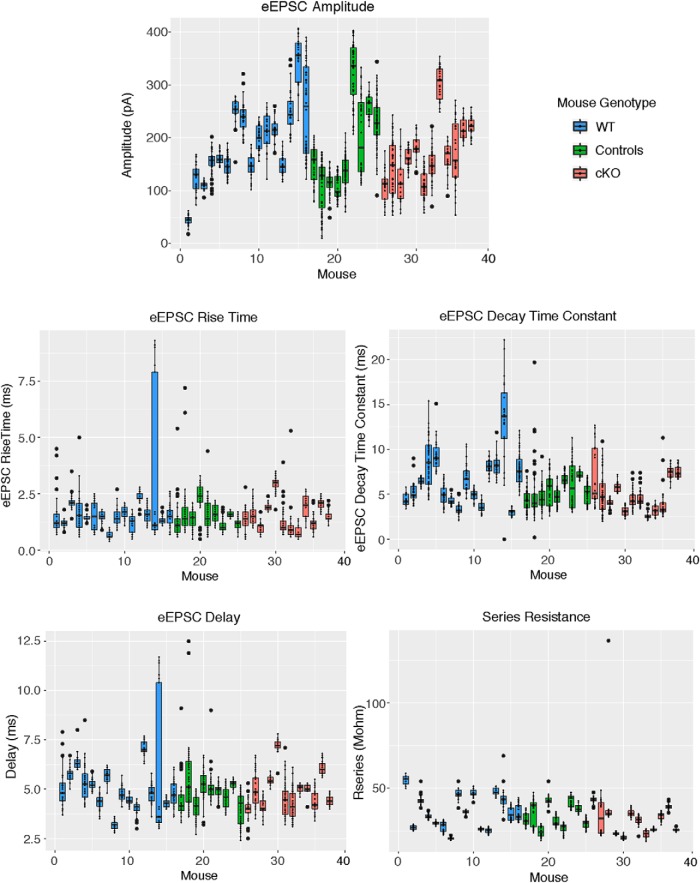
Plots showing all the data points for each of the eEPCS parameters analyzed. The small black circles are the individual data points, the horizontal lines indicate the 25th quartile, the median, and the 75th quartile. The large black circles indicate data points lying outside 1.5 times the interquartile range. The whiskers indicate the upper and lower values.

SCN neurons which were characterized by large maximal eEPSC amplitude were analyzed further and the parameters of these eEPSCs were compared between the four mouse genotypes: WT (mice/neurons; 16/19); littermate controls (9/20); cKOs (12/16); and dKOs (2/3). During the whole cell recordings, the series resistances (MΩ) were similar among the mouse genotypes: WT 36.3 (95% confidence interval: 31.9–41.4), littermate controls (controls), 33.7 (95% confidence interval: 29.8–38.0), and cKO 31.0 (confidence interval: 27.4–35.0; GEE analysis, *p* = 0.215). There were no significant differences in the eEPSC peak amplitude, time to peak, rise time, or decay time constant (for values and statistical analysis, see Table 1). The stimulus voltages needed to evoke the threshold eEPSC and the maximal amplitude EPSC were not significantly different between mouse groups (GEE analysis, *p* = 0.569 and *p* = 0.065, respectively; Table 1). Data for individual mice are presented in Figure 7. The data for dKO mice were excluded from statistical analysis because of a small number of animals and recorded neurons. The recorded eEPSC parameters for the dKO mice (*n* = 3 cells) were in similar ranges for the maximal eEPSC amplitude (83.2–248.4 pA), time-to peak (3.60–4.96 ms), rise time (0.83–1.75 ms), and decay time constant (2.95–6.48 ms). This demonstrates that some ipRGCs in cKO animals remained capable of releasing synaptic vesicles loaded with glutamate onto SCN neurons.

**Figure 8.**
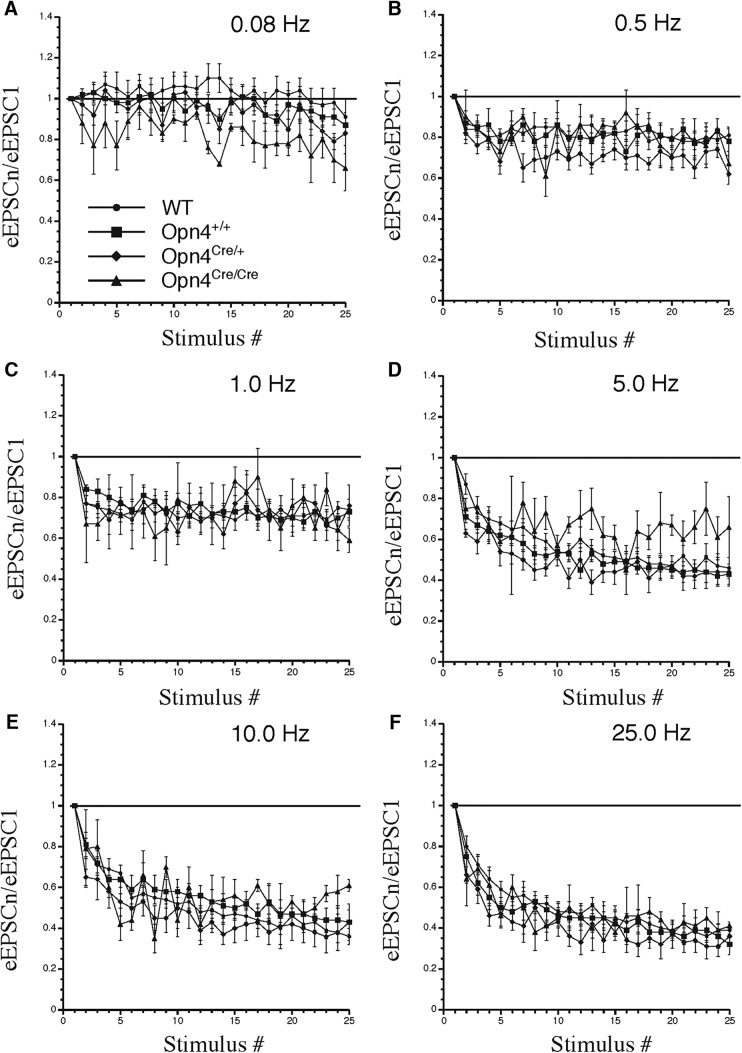
Short-term synaptic depression in SCN neurons during stimulus train application. Stimulation of the optic chiasm with trains of 25 stimuli at 0.08- to 25-Hz frequencies (***A–F***). The amplitude of each subsequent eEPSC in the train was normalized to the amplitude of the first eEPSC: eEPSCn/eEPSC_1_. Frequency-dependence changes of eEPSC amplitude in WT (*n* = 18 neurons), control (Opn4^+/+^; Vglut2^loxP/loxP^; *n* = 16 neurons), cKO (Opn4^Cre/+^; Vglut2^loxP/loxP^; *n* = 8 neurons), and dKO (Opn4^Cre/Cre^; Vglut2^loxP/loxP^; *n* = 2 neurons) mice during stimulation with frequencies ranging from 0.08 to 25 Hz.

We further hypothesized that the rate of synaptic vesicle replenishment with glutamate in RHT terminals might be altered during long repetitive stimulation of the optic chiasm, which can exhaust the synaptic vesicle replenishment and glutamate release in vGlut2 KO mice. We also expected that in vGlut2 KO mice synaptic vesicle replenishment might not compensate the synaptic vesicle depletion and, therefore, the steady-state eEPSC amplitude during short-term synaptic depression will be significantly lower than in WT control mice. To investigate this, the optic chiasm was stimulated with trains of stimuli, which induced synaptic depression (Moldavan and Allen, 2010; Moldavan et al., 2013). In the four mouse groups WT, littermate controls, cKO, and dKO, repetitive 0.08-Hz stimulation did not induce synaptic depression (Fig. 8*A*), which was observed only at higher stimulus frequencies: 0.5, 1, 5, 10, and 25 Hz (Fig. 8*B–F*). During application of subsequent stimuli in the train, there was a frequency-dependent decrease in the eEPSC amplitude which reached a steady-state value and was not significantly different among the four mouse groups.

We hypothesized that vGlut2 KO could increase the number of failures in glutamate release from RHT terminals. To evoke EPSCs the optic chiasm was stimulated at 0.08 Hz with a stimulus strength 1.5–2.0 times the threshold. Under these experimental conditions the stimulation did not induce synaptic depression and faithfully evoked EPSCs without failures in mice in all genotype groups. Therefore, to check whether the vGlut2 KO affects the variability of the eEPSC amplitude, we measured the SD of the eEPSC amplitude of each group of animals. Coefficient of dispersion [coefficient of variation (CV)] for each group was estimated as a ratio of SD to the mean eEPSC amplitude (CV = SD/mean): WT (0.15), controls (0.23), and cKO (0.21). Application of the Kruskal–Wallis rank sum test did not reveal significant differences between the groups (*p* = 0.101).

### Spontaneous EPSCs in cKO mice

The amplitude, decay time constant (τ), charge transfer (pA/ms), and the frequency of sEPSCs were not different among the mouse groups (Table 2). The rise time significantly decreased in the littermate control mice compared with WT mice but were not different between cKO and Controls/WT (one–way ANOVA followed by Tukey HSD test, *F*_crit_ = 3.18, *F*_(2,51)_ = 4.11, *p* < 0.02; Table 2). The data range for dKO mice (*n* = 2 cells) were: sEPSC amplitude (15.2–26.4 pA), rise time (1.1–3.0 ms), decay time constant (3.2–3.7 ms), frequency (0.1–6.7 sEPSC/s), and area (charge transfer: 66.7–73.7 pA/ms). sEPSCs were mediated by AMPA receptors as they were blocked by CNQX (20 µM).

**Table 2.** Parameters of spontaneous EPSCs in SCN neurons

Genotype	Mice (*n*)	Neurons (*n*)	Amplitude (pA)	Rise time (10–90% of amplitude) (ms)	Decay time constant (ms)	Area (charge) (pAm)	Frequency events/s
WT	10	28	14.4 ± 0.9	1.9 ± 0.1	2.9 ± 0.2	46.2 ± 4.6	2.6 ± 0.8
Control	3	11	17.1 ± 1.7	1.4 ± 0.2	3.6 ± 0.2	43.9 ± 4.0	1.9 ± 0.7
cKO	10	15	16.3 ± 1.1	1.6 ± 0.2	2.9 ± 0.2	46.3 ± 3.4	2.4 ± 0.7
ANOVA F-crit			3.18	3.18	3.18	3.18	3.18
ANOVA *F*_(2,51)_			1.44	4.11	0.55	0.06	0.12
p, (cKO vs WT)			0.47	0.21	0.95	1.00	0.99
p, (cKO vs control)			0.91	0.53	0.78	0.95	0.92
p, (control vs WT)			0.29	0.02*	0.56	0.95	0.86

WT: wild-type controls; control: littermate controls (Opn4^+/+^); cKO: Opn4^Cre/+^; Vglut2^loxP/loxP^. *p < 0.05, one-way ANOVA followed by Tukey HSD *post hoc* test (Excel, Igor Pro). MiniAnalysis software (Synaptosoft Inc.) was used for acquisition and analysis of spontaneous EPSCs parameters.

## Discussion

Genetically modified mice have been critical to furthering our understanding of virtually every facet of biology including providing invaluable insights into the molecular regulation of sleep and circadian rhythms (Lowrey and Takahashi, 2011; Funato et al., 2016). However, the power of genetic mouse models is dependent on physiologic verification of the intended genetic modifications. In the current study, we generated two independent but similar strains of mice in which the glutamate transporter vGlut2 was selectively knocked out in ipRGCs using Cre-lox recombination with the aim of eliminating glutamatergic retinal input to the SCN. To evaluate functionally the effect of knocking out ipRGC glutamatergic innervation of the SCN, circadian behavior was examined under several lighting conditions in a series of experiments. It was anticipated that in these experiments any observed residual circadian behavioral responses to light could be attributed to the remaining RHT peptidergic input to the SCN (Hannibal, 2006; Tsuji et al., 2017).

To physiologically verify that the genetic modification strategy employed to eliminate RHT glutamatergic signaling was successful, electrophysiological recordings were made from SCN neurons in a hypothalamic slice preparation from animals whose circadian behavior had been evaluated. To our surprise, all vGlut2 cKO and dKO animals recorded retained RHT glutamatergic signaling. Although a slightly stronger stimulus was required to induce maximal amplitude EPSCs in cKO animals compared to controls, this difference (16.7 vs 14.3 V) did not reach statistical significance (*p* = 0.065; Table 1). Since RHT glutamatergic input was not completely eliminated in ipRGC vGlut2 cKOs, circadian behavioral responses to light could not be assigned solely to ipRGC peptide neurotransmitters.

There are several potential explanations for the observed ipRGC glutamatergic input to the SCN in ipRGC vGlut2 cKO animals. However, the simplest explanation is that Cre-recombinase was not expressed (or insufficiently expressed) in at least some ipRGCs afferent to the SCN and consequently not all ipRGC glutamatergic transmission was eliminated when Opn4^Cre^ mice were crossed with vGlut2^loxP^ animals. Indeed, Cre-recombinase was not expressed in all ipRGCs in the Hopkins Opn4^Cre^ mouse line used herein and by several other laboratories to generate ipRGC vGlut2 cKOs (Delwig et al., 2013; Purrier et al., 2014; Gompf et al., 2015; Keenan et al., 2016) or other cKOs (Chew et al., 2017). Based on melanopsin immunostaining, it was indicated that “the majority of melanopsin-immunoreactive cells expressed the reporter proteins” in which Cre-mediated recombination activated the expression of EGFP (Ecker et al., 2010). No further quantification was provided. In the other Opn4^Cre^ mouse line we used to generate ipRGC vGlut2 cKOs (Salk-Cre mouse line), Hatori and coworkers indicated that a “small number of RGCs stained positive for melanopsin, but showed no detectable level of EGFP fluorescence” (Hatori et al., 2008), qualitatively similar to the Hopkins-Cre mouse line. It was offered that the disparity between EGFP expression and melanopsin immunostaining might represent ipRGCs with insufficient Cre expression, Cre activity, or EGFP level. It would appear based on our observations that at least a small but functionally significant fraction of melanopsin-expressing ipRGCs afferent to the SCN do not express or insufficiently express Cre-recombinase in the two Opn4^Cre^ mouse lines evaluated in this study. Thus, the ipRGCs lacking Cre-recombinase expression continue to use vGlut2 to load glutamate into synaptic vesicles and provide light-evoked glutamatergic input to the SCN. There is no doubt that the eEPSCs observed in the SCN in this study were glutamatergic since all evoked responses were completely blocked by the application of CNQX, a potent and selective AMPA/kainite receptor antagonist. Using a similar recombination approach, when vGlut2 is knocked out in vGlut2-expressing neurons, glutamatergic synaptic transmission is eliminated (Hnasko et al., 2010; Koch et al., 2011). These findings are consistent with our interpretation that vGlut2 expression was not eliminated in all ipRGCs afferent to the SCN. It is not known why vGlut2 expression was retained in some ipRGCs, but the vagaries of conditional gene targeting are well documented (Schmidt-Supprian and Rajewsky, 2007).

If only a small number of ipRGCs in the cKOs maintained glutamatergic input to the SCN, then in the majority of ipRGCs vGlut2-mediated glutamatergic transmission was eliminated as anticipated. This interpretation is supported by the previously reported loss of vGlut2 expression in melanopsin-expressing RGCs in cKO animals although the extent of the loss of vGlut2 expression in these studies was not quantifiable due to technical limitations (Delwig et al., 2013; Purrier et al., 2014). Additional support for a large reduction in RHT glutamatergic input to the SCN in vGlut2 cKO animals was the observed altered circadian behavioral responses to light (current study; Purrier et al., 2014; Gompf et al., 2015). However, the extent to which cKO animals showed abnormal behavioral responses to light was highly variable in this study and in others (Delwig et al., 2013; Purrier et al., 2014; Gompf et al., 2015) and entrainment was light intensity related. For example, in our study and in the report of Gompf et al. (2015), some cKO animals showed very little response to light essentially free-running throughout the different lighting conditions (Figs. 1*C*, 4*H* ), whereas other cKO animals showed relatively normal entrainment (Figs. 1*J*, 4*B* ; Gompf et al., 2015).

The varying responses of ipRGC vGlut2 cKO animals to light may reflect differences among animals in: (1) the total number of ipRGCs that continued to transmit glutamatergic signals to the SCN; (2) the specific subtype of ipRGC (M1 or M2; Baver et al., 2008) that continued transmitting glutamatergic signals to the SCN; and/or (3) the specific region of the SCN that the glutamatergic transmitting ipRGCs targeted (Fernandez et al., 2016). There is some information regarding the number of ipRGCs needed to mediate photoentrainment. When >90–95% of ipRGCs are ablated, which results in only very few retinal afferents to the SCN, mice no longer entrain to a 12L (150 lux):12D (0 lux) cycle (Hatori et al., 2008). Entrainment can be improved or restored to ipRGC vGlut2 cKO (presumably with only a small number of glutamatergic transmitting ipRGCs) under bright light conditions (1000 lux, current study; 2000 lux; Gompf et al., 2015) albeit with an abnormal phase angle.

We hypothesized that knocking out Vglut2 in ipRGCs would eliminate glutamatergic neurotransmission in the RHT by preventing glutamate from being loaded into synaptic vesicles. If our hypothesis were correct, we then predicted that stimulation of the optic chiasm would not evoke the EPSCs typically observed in SCN neurons (Kim and Dudek, 1991; Jiang et al., 1997; Moldavan et al., 2006; Moldavan and Allen, 2010, 2013). However, stimulation of the optic chiasm did evoke glutamatergic EPSPs and several parameters of the eEPSCs (the peak amplitude, the time-to-peak, and the rise time) were similar among all mice. Stimulus voltages applied to evoke the threshold eEPSC and the maximal amplitude EPSC were not significantly different between mouse groups. No failures of eEPSC at applied stimulus conditions were observed in any mouse, and no significant changes of eEPSC amplitude deviation were observed between cKO mice and controls. Also, a slightly stronger stimulation of the optic chiasm was needed to evoke a maximal amplitude EPSC in vGlut2 cKO animals compared to control mice (*p* = 0.065), suggesting slightly altered evoked glutamate release in cKO animals. Thus, during application of single stimuli, the synaptic vesicles in the RHT axonal terminals of cKO mice apparently contained sufficient glutamate to maintain synaptic transmission but the number of vesicles being released was reduced in cKO animals.

Although it appears that the residual RHT glutamatergic neurotransmission we observed in vGlut2 cKO animals in the current study was the result of some ipRGCs retaining the ability to load glutamate into synaptic vesicles using vGlut2, there are other possibilities that deserve mentioning. In the absence of vGlut2 the possibility exists that vGlut1 and/or vGlut3 were upregulated allowing glutamate to be loaded into synaptic vesicles in at least some ipRGCs resulting in the glutamatergic responses we observed following stimulation of the optic chiasm. Purrier et al. (2014) addressed the question of compensatory expression of vGlut1 and vGlut3 in ipRGCs and found no evidence to support this possibility. However, vGlut expression in RGC somas is not robust and if upregulation occurred in only a small number of ipRGCs afferent to the SCN this would have been very difficult to detect. Because of the low expression levels in the somas of RGCs, vGlut2 in ipRGC axon terminals in the SCN has also been examined with a small decrease detected in cKO mice (Delwig et al., 2013). Other glutamatergic inputs to the SCN (Pickard, 1982) make it difficult to assess either a decrease in vGlut2 in ipRGCs axon terminals in the SCN or potential compensatory expression of other glutamate transporters especially if this occurs in only some ipRGCs. Another possibility is that conventional RGCs that do not typically innervate the SCN send axonal branches into the SCN of vGlut2 cKO animals. This possibility was also addressed by Purrier et al. (2014) and the RHT innervation of the SCN appeared normal, although a small number of RGCs aberrantly innervating the SCN would also be very difficult to detect. It is important to emphasize that in either the case of compensatory ipRGCs expression of vGlut1 and/or vGlut3 or aberrant RGC projections to the SCN in vGlut2 cKO animals, the conclusion of our study, that residual SCN-mediated behavioral responses to light cannot be solely attributed to RHT PACAP afferents to the SCN, would not change.

In both WT control and cKO mice, there were broadly two types of response in SCN neurons to increased stimulation voltage: some neurons showed a gradual increase in eEPSC amplitude whereas other showed a rapid increase to maximal amplitude with only a 1.5- to 2.0-times increase in stimulation strength over threshold. It would appear that different populations of SCN neurons integrate RHT input differently. This observation is interesting since it was recently reported that the spike output of M1 ipRGCs afferent to the SCN also breaks down into two types: those that show a monotonic increase in firing with increasing irradiance and those that showed an increase in firing rate with a subsequent sharp drop in firing rate as irradiance increased (Milner and Do, 2017). It will be interesting to determine how M1 ipRGCs with different irradiance-firing relations are integrated in the SCN and how different SCN neurons integrate this input.

Taken together, our recordings of evoked and spontaneous EPSCs indicate that significant glutamatergic neurotransmission remains in RHT afferent fibers innervating the SCN of the cKO mice and that the KO model resulted in only subtle changes in the rate of vesicular replenishment with glutamate even at high stimulation frequencies. These results are consistent with the behavioral data observed in this study and other studies using the ipRGC vGlut2 cKO mouse. Unfortunately, the residual RHT glutamatergic transmission in the cKO mouse model limits the usefulness of this model for examining the role of RHT peptidergic afferents to the SCN.

## Synthesis

Reviewing Editor: Quentin Pittman, University of Calgary Decisions are customarily a result of the Reviewing Editor and the peer reviewers coming together and discussing their recommendations until a consensus is reached. When revisions are invited, a fact-based synthesis statement explaining their decision and outlining what is needed to prepare a revision will be listed below. The following reviewer(s) agreed to reveal their identity: R. Lane Brown

Both reviewers agree that your submission reports important data that may very well necessitate a reconsideration of the results from previous KO studies that had previously been ascribed to PACAP. The reviewers have also made suggestions for improvement of your manuscript.

The most significant of these, which was brought up by both reviewers, is to provide further information concerning the possibility of upregulation of other vGluts. Immunohistochemistry has been suggested as the most appropriate means to do so.

Both reviewers also have suggested that you are missing an opportunity to correlate better the behavior with the electrophysiology and if your ‘n’ is adequate to do so you are urged to consider this. The success rate of the electrophysiological recordings should also be given.

A third major point is that some of the statistics should be revisited as suggested.

In addition, the reviewers have raised a number of other questions and provided suggestions that you may want to consider. Both reviews are copied below and you should consult them carefully in making your revisions:

Reviewer 1:

This is an important finding, and will necessitate a reconsideration of results from other opn4-Cre/Cre mediated KO studies, which ascribed residual function to signaling via PACAP. It also provides a cautionary tale for making assumptions about the efficiency of conditional genetic deletions.

Overall, the paper was well-written and the experiments were quite convincing, but the significance of this finding would be enhanced if the authors could address a few further questions:

1. Is it possible that residual glutamatergic transmission in the SCN is due to compensatory expression of other glutamate transporters? Although previous studies have shown that vGlut2 is absent in the cell bodies of (selected) ipRGCs in the Opn4-Cre/+;vGlut2-fl/fl animals, and that there is no compensatory expression of vGlut1 or vGlut3, it would be hard to see if a relatively small, but significant, fraction of the ipRGCs were exceptions. Perhaps a comparison of immunostaining of the SCN for vGlut1, vGlut2, and vGlut3 in WT and cKO animals would be more sensitive and informative. Bilateral enucleation has been reported to dramatically reduce vGlut2 expression in the SCN (Fujiyama et al., 2003).

2. Since it appears that behavioral studies and electrophysiological studies were performed on the same animals, do the authors have sufficient statistical power to correlate the strength of circadian entrainment with the strength of glutamatergic transmission? Did every SCN neuron from which a successful recording was obtained respond to RHT stimulation? If not, was there any significant difference between WT and cKO animals? Were recordings performed on and Opn4-Cre/Cre;Vglut2-fl/fl animals, which might be expected to have enhanced Cre activity?

3. Is there any way to definitely determine if the glutamatergic response in the cKO is due to synaptic terminals of ipRGCs, or is it possible that axons from “regular” RGCs have infiltrated the SCN?

4. It would be interesting to confirm that the glutamatergic responses are actually vGlut-dependent. Are there any pharmacological blockers of vGlut that could be tested in the electrophysiology experiments? A quick perusal of the literature suggests that useful reagents targeting vGluts are severely lacking.

Minor comments:

1. The “Hnasko” reference is missing the title of the article.

2. Lux numbers are sometimes missing in some figure panels.

Reviewer 2:

This well-written article is an important examination of the claim that residual entrainment in some mice with selective loss of vesicular glutamate transporter 2 in ipRGCs expressing Opn4 is due to other signaling molecules such as PACAP. Surprisingly, the present results indicated that these mice do still exhibit glutamatergic transmission to SCN neurons, as measured by evoked EPSCs via optic nerve stimulation. These EPSCs are blocked by AMPA receptor antagonists and also have similar properties in all genotypes compared. The investigators have done an outstanding job of using stimulation response curves (intensity and trains) to ascertain properties such as glutamatergic synaptic vesicle availability and replenishment. The authors also present long-term circadian behavioral phenotyping on the mice they studied; however, the behavioral phenotypes were not linked to the physiology. This last point seems to be a missed opportunity. While others have linked SCN molecular clock gene rhythmicity to individual differences in behavior and entrainment (see PMID: 26108632), very few attempts have been made to link physiological responses to behavior. Given the tremendous variability in entrainment phenotypes (even in the control groups), this appears to be a missed opportunity. Other concerns are listed below:

1. Opn4Cre/+;vGlut2+/+ animals would allow investigators to determine if the effect of Opn4Cre alone.

2. A better description of the validation of this animal model in prior work would help. Validation appears to be limited to confirmation that the Cre is expressed in ipRGCs but was there any validation of knockdown of vGlut2 expression itself (via either double label IHC or fluorescent ISH)? If this has not been done in prior studies, it should be done here and if it has, it should be more explicitly described including the percentage vGlut2 knockdown.

3. The behavioral results are very descriptive, likely due to the small sample size. The variability in tau in even the control mice is huge. Were the same exact days in DD used for analysis in all animals? In Experiment 1, the littermate controls are almost all female and one mouse had activity onsets that were not analyzable (was this a male or female?). Thus caution should be given to directly comparing tau of the cKO mice with the controls since great differences in the number of mice from each sex are included. If none of these animals were used in the physiology experiments later, this should be clearly stated.

4. The behavior in Experiment 2 should be quantified in terms of the onset error or phase angle of entrainment (and daily error) for each mouse in the last week of the LD cycle (before sacrifice). This information could then be used to correlate with the physiology data from that animal. The same should be done for the animals used in Experiment 3.

5. For the RHT-evoked EPSCs, it is important to also report the success rate of the recordings. In Table 1, recordings from control mice were much more successful than WT, cKO or dKO mice, which resulted in ~ 1 recorded cell/animal. Were there times in which stimulation did not evoke an EPSC and if so, were those data included in the results somehow? In the sEPSCs (Table 2), it is strange that the WT and control mice appear to be quite different in some of the parameters. Is there an explanation for this?

6. All statistical analyses and stats (t values, F values, etc) should be reported. Other statistical analysis issues:

a. The comparison of threshold distributions (Fig. 5E) should not analyzed with a t-test - this will only tell you if the means are different. Differences in the shape of a distribution should be made with a Kolmogorov-Smirnov test, or something similar. The variability in these data could be used to correlate with behavioral phenotype.

b. It is surprising that the curves in Fig. 6E,F were not different statistically, especially at the higher voltages. These data should be analyzed with a two-way RM-ANOVA.

c. In Table 1, there are four groups (although dKO should probably not be included due to the low sample size) and thus an ANOVA followed by post hoc tests is more appropriate, for all parameters in the table. It is unclear why cKO are only compared to WT and not Controls with a t-test.

d. In Table 2, a one-way ANOVA is used; however, dKO should be excluded because there is an n of 2. It is okay to keep this row in the table for descriptive purposes, but this group cannot be included in any analysis. The asterisks in this table should indicate the outcomes of post hoc tests, rather than ANOVA.
